# Successful conception in a 34-year-old lupus patient following spontaneous pregnancy after autotransplantation of cryopreserved ovarian tissue

**DOI:** 10.1177/0961203319839482

**Published:** 2019-03-24

**Authors:** G Chehab, J Krüssel, T Fehm, R Fischer-Betz, M Schneider, A Germeyer, M B Suerdieck, V Kreuzer, J Liebenthron

**Affiliations:** 1 Policlinic of Rheumatology and Hiller Research Unit Rheumatology, Heinrich-Heine-University, Düsseldorf, Germany; 2 Department of Obstetrics/Gynecology and Reproductive Endocrinology and Infertility (UniKiD), Heinrich-Heine-University, Düsseldorf, Germany; 3 UniCareD, University Cryobank for Assisted Reproductive Medicine and Fertility Protection at UniKiD Düsseldorf, Heinrich-Heine-University, Düsseldorf, Germany; 4 Department of Obstetrics and Gynecology, Heinrich-Heine-University, Düsseldorf, Germany; 5 Department of Gynecological Endocrinology and Fertility Disorders, University Women's Hospital, Heidelberg, Germany; 6 Gyn-A.R.T.AG, Zurich, Switzerland.

**Keywords:** Pregnancy, renal lupus, systemic lupus erythematosus

## Abstract

Premature gonadal failure is a common problem in patients with systemic lupus erythematosus (SLE) when gonadotoxic therapies are applied. The preservation of gonadal function and fertility is of great importance to many predominantly young SLE patients. Some fertility preservation methods are well established and well known, whereas others are considered more cautiously. In particular, the cryopreservation of ovarian tissue is a rarely chosen fertility preservation option for SLE patients of (pre)fertile age. We report the first case of successful conception and pregnancy of an SLE patient after autotransplantation of cryopreserved ovarian tissue. A 26-year-old SLE patient decided to undergo cryopreservation of ovarian tissue when receiving cyclophosphamide for lupus nephritis. Tissue removal, preparation, cryopreservation and quality control was performed, as described, according to current state-of-the-art techniques. After 6 years of being in remission using azathioprine and belimumab, her ovarian tissue was autotransplanted because of premature ovarian failure, diagnosed at the age of 32, and a wish to conceive. She conceived spontaneously 8 months later, having a diamniotic-dichoriotic twin pregnancy. The children were born prematurely due to preterm premature rupture of membranes in the 32nd week of gestation; mother and children are doing very well 8 months later. We regard the procedure to be an option worth consideration for our predominantly young SLE patients.

## Introduction

Premature gonadal failure is a common problem in patients with systemic lupus erythematosus (SLE) and exposure to cyclophosphamide.^1–3^ Although innovative therapeutic regimes with lower cumulative doses (Euro-Lupus protocol) promise less detrimental effects on gonadal function,^4^ prevention of gonadal toxicity, and preservation of gonadal function and fertility, remain of great importance to predominantly young SLE patients. There are several options to preserve fertility in such patients where it is not possible to avoid the use of medication impairing fertility (especially cyclophosphamide). Commonly known options are the administration of gonadotropin-releasing hormone (GnRH) agonists and the cryopreservation of oocytes. A less well-known option is the cryopreservation of ovarian tissue.^5^ In German-speaking countries (Germany, Switzerland and Austria), the *Ferti*PROTEKT network register (www.fertiprotekt.com) has collected data on therapies and the effectiveness of fertility preservation measures since 2007. Only three SLE patients with cryopreserved ovarian tissue have been transplanted so far (one patient twice) (official data request from the registry by the authors). Although other publications have reported cryopreservation of ovarian tissue to be an option for fertility preservation in non-malignant diseases (including SLE),^6,7^ to the best of the authors' knowledge, there have been no reports of successful conception or pregnancy of an SLE patient after autotransplantation of cryopreserved ovarian tissue until now.

## Case

We present the case of a young woman who was diagnosed with SLE at the age of 17, based on the presence of polyarthritis, pleuritis, oral ulcers, haemolytic anaemia, leukopenia, positive antinuclear antibodies and elevated anti-dsDNA. Medical treatment with prednisolone, hydroxychloroquine (HCQ) and azathioprine was started. During the course of her disease, a cerebellar infarction occurred that fortunately did not result in physical or cognitive limitations. Antiphospholipid screening was persistently negative (including anticardiolipin antibodies, anti-β2-glycoprotein I antibodies and lupus anticoagulant). She received long-term treatment with low-dose aspirin afterwards. At the age of 20, an unplanned pregnancy was terminated due to active disease and lack of a permanent partnership. By 2009, at the age of 25, anti-dsDNA titres had risen sharply with persistent low complement levels, 3 + blood 4 + protein on urine dipstick and a urine protein–creatinine ratio of 2039 mg/gC (<150). A renal biopsy showed class II/V lupus nephritis. She was started on treatment with mycophenolate mofetil (MMF), which was found to be insufficiently effective. Therefore, it was decided in May 2010 to switch treatment to cyclophosphamide based on the protocol of the National Institute of Health trial from 1992.

With regard to the preservation of gonadal function, she was referred to the Department of Obstetrics/Gynecology and Reproductive Endocrinology and Infertility (UniKiD) for counselling. The available options (controlled ovarian stimulation, retrieval of mature metaphase-II oocytes and cryostorage for future use in intracytoplasmic sperm injection-therapy, and laparoscopic removal of ovarian tissue followed by cryostorage of ovarian cortex for future use as transplant) were extensively discussed. Since she had already received four cycles of cyclophosphamide, ovarian cryopreservation was recommended and the patient decided to have ovarian tissue removed. At that time, she had received four therapy cycles of cyclophosphamide (cumulative dose 2.1 g) and reported that the previously regular menstrual cycle (28–30/5) had become irregular (6–7 weeks); gonadotropins were within the normal range (luteinizing hormone [LH] 3.2 mIU/ml and follicle-stimulating hormone [FSH] 5.0 mIU/ml).

## Laparoscopic removal of ovarian tissue, tissue preparation, cryopreservation and first quality control test

**Figure 1:**
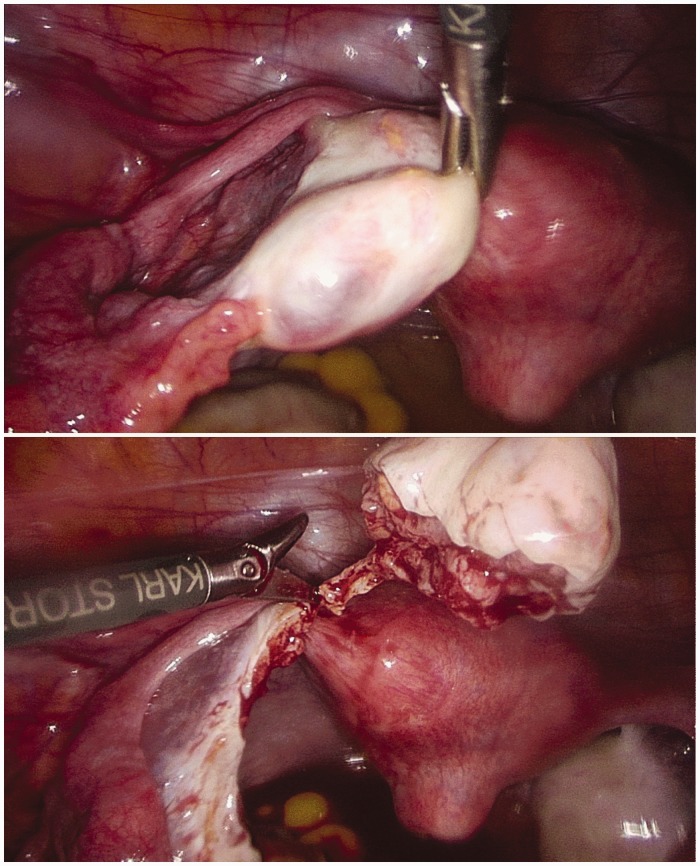
Image of laparoscopic removal of ovarian tissue (two-thirds of the right ovary).

In September 2010, the patient underwent laparoscopy at the university's department of obstetrics and gynaecology Duesseldorf. Two-thirds of her right ovary were removed with non-cauterizing scissors (Figure 1) and immediately extracted from the abdomen. The patient was released from the hospital 4 hours after the operation. A detailed description of the process of tissue removal, transportation, tissue preparation, cryopreservation protocol and quality control is provided in the supplementary material for this article.

From the removed tissue, 12 ovarian cortex stripes (∼8 × 4 × 1 mm) were prepared for later transplantation and cryopreserved. From the remaining cortical tissue, six small standardized biopsies were obtained from different areas of the prepared cortex for quality control. Cryopreservation of ovarian tissue was performed by a slow-freezing protocol modified from the procedure initially described by Gosden in 1994 (see supplement).

**Figure 2:**
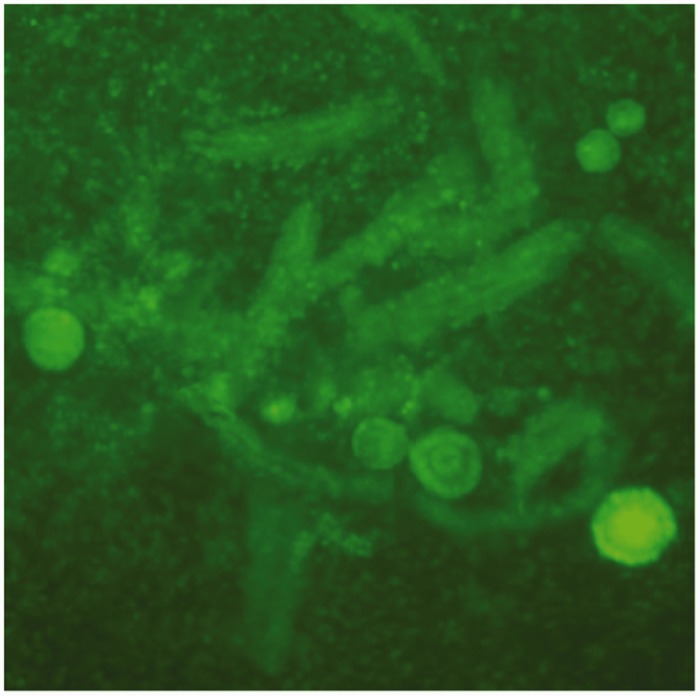
Viable follicles of 3 × 2 mm thawed/digested ovarian cortex biopsies as viewed under a fluorescence microscope. Whole follicle number/mean value of 3 × 2 mm biopsies in digest: 68.

Three 2-mm diameter biopsies were used for quality analysis of the density and viability of primordial and primary follicles embedded in the cortical tissue directly after transportation, preparation and before cryopreservation, and three after thawing of the cryopreserved control biopsies before transplantation (thawing sample)^8,9^ (Figure 2). After counting of the freshly prepared biopsy, before cryopreservation, 74 viable primordial follicles (PROFs) could be determined and, after cryopreservation (6 years later), in the same amount of tissue (three 2-mm biopsies) 68 PROFs were observed.

In 2012, after a prolonged time of 21 months, remission was attained (cumulative cyclophosphamide dose of 10.9 g) and a switch to MMF for maintenance therapy was made. In October 2014, approximately 5 years after her first renal flare, a relapse of her lupus nephritis (class IV/V) occurred despite the use of MMF. Again, she was treated with cyclophosphamide, this time according to the Euro-Lupus trial protocol, resulting in an overall cumulative cyclophosphamide dose of 13.9 g. Subsequent remission maintenance was initiated using azathioprine and belimumab in early 2015. In mid-2015, being persistently in remission (clinical remission on treatment according to the new definitions of remission in SLE^10^) she again expressed a desire to have a child. After extensive discussion of the risks of pregnancy with regard to her condition and medication, she decided to continue her current immunosuppressive therapy and try to become pregnant.

In September 2016, the patient was referred again to UniKiD to discuss her fertility options. She reported secondary amenorrhea, hormone levels were menopausal (FSH 97.1 mIU/ml, LH 54.2 mIU/ml, oestradiol 38 pg/ml and anti-Müllerian hormone (AMH) < 0.03 µg/L) and transvaginal ultrasound revealed no sign of follicular activity (antral follicle count [AFC] = 0). She also suffered from hot flushes. An andrological examination of the husband’s semen revealed normozoospermia (World Health Organization 2010 criteria). Due to her premature ovarian failure, it was decided to transplant part of the cryopreserved ovarian tissue.

## Second quality control including test of cryopreserved/ovarian cortex reserve and autotransplantation

According to Liebenthron et al.^9^ and Beckmann et al.^8^, follicle density (mean from fresh and thawed samples: 71 PROFs) as well as serum AMH concentrations 1.7 ng/ml, FSH: 5.0 mIU/ml and the patient's age at tissue removal (26 years) were regarded as the ovarian reserve parameters, and were used to calculate the amount of ovarian tissue required for transplantation. We decided to transplant four pieces sized 4 × 8 × 1 mm^3^ into one peritoneal pocket near the fallopian tube. The thawing protocol was modified from published procedures (see supplement).

**Figure 3:**
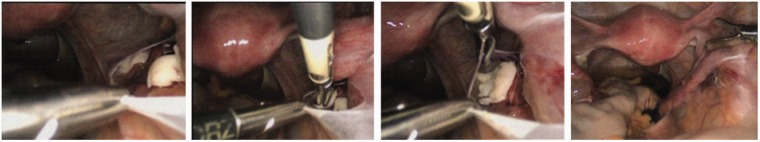
Example image of autotransplantation of ovarian tissue into a left small peritoneal pocket.

In October 2016 the laparoscopy was performed. Tubal patency by chromopertubation was positive only on the right fallopian tube, so the tissue was transplanted into a small peritoneal pocket (2 × 3 cm) that was prepared dorsal from the ligamentum infundibulopelvicum into the right pelvic wall (Figure 3). Four pieces of previously cryopreserved and thawed ovarian tissue were placed side-by-side without overlap into the peritoneal pocket, with the ovarian surface oriented to the backside of the pelvic peritoneum. The pocket was closed and no suture was applied.

Seven weeks after transplantation (December 2016), the patient was seen for a first follow-up visit at UniKiD. She reported a significant reduction of hot flushes and hormone levels showed signs of ovarian tissue activity (oestradiol 208 pg/ml and LH 48.5 mIU/ml). Transvaginal ultrasound showed an endometrial thickness of 12 mm and one antral follicle was visible in the right pelvic wall at the site of the transplant. Ten-days later she had menstrual bleeding, so it was assumed that she had had a spontaneous ovulation in mid-December.

In February and March 2017, the patient was seen for follow-ups. Serum gonadotropins had reverted back to premenopausal levels (FSH 7.6 mIU/ml and LH 1.9 mIU/ml). Menstrual cycle lengths were 20 and 23 days. Three-months later she conceived spontaneously and was subsequently diagnosed as having a diamniotic-dichoriotic twin pregnancy 1 week after the last belimumab dose, which was discontinued afterwards. Her SLE Disease Activity Index 2000 score was 4 (only due to low-complement and increased DNA binding), with normal blood pressure and urine–protein creatinine ratio (146.5 mg/gC (<150)). Due to her medical history and clinical characteristics, her pregnancy was classified as high-risk, following the European League Against Rheumatism recommendations for pregnancies.^11^ Correspondingly, tight medical supervision was initiated (monthly checks with rheumatologists and bi-weekly visits to gynaecologists including additional ultrasound examinations).

Lupus-specific treatment with HCQ, azathioprine and low-dose prednisolone (5 mg/day), as well as folic acid and oral iron, were continued during pregnancy. Due to the previous cerebellar infarction, our patient received long-term treatment with low-dose aspirin. In view of the increased thromboembolic risk during pregnancy, renal involvement and twin pregnancy, we decided, after interdisciplinary discussion, to add low-molecular weight heparin despite negative antiphospholipid antibodies in this special situation.

Treatment with a renoprotective angiotensin II receptor blocker (ARB) was discontinued because of its fetal toxicity, resulting in a low increase of proteinuria without signs of active lupus nephritis. First-trimester ultrasound in week 12 and detailed organ ultrasound in week 22 including Doppler sonography revealed normal fetal growth without any pathological abnormalities. SLE remained stable; however, during the second trimester, her iron deficiency anaemia was aggravated. By the 26th week of gestation she was hospitalized with successively occurring tachycardia, rising blood pressure and a temporary febrile episode. Preeclampsia or HELLP (hemolysis, elevated liver enzymes, and low platelet count) syndrome could be ruled out. Additional medical therapy was not required.

In January 2018, her two baby sons were delivered in the 32nd week of gestation by caesarean section due to preterm premature rupture of membranes (PROM). Three- and 6-months later, the mother and babies present themselves in a good overall condition. Only umbilical hernias were detected in both infants. A reappearance of proteinuria in the mother (up to 3 g/day), without signs of active urine sediment, has improved after restarting ARB treatment.

## Conclusion

In addition to the more or less well-established known measures to preserve fertility in SLE patients (substitution of GnRH and cryopreservation of oocytes), we present a case of successful conception after autotransplantation of cryopreserved ovarian tissue.

The method of cryopreservation of ovarian tissue, which was still regarded as experimental in the past, is now established and recognised in postpubertal and adult fertile patients.^8,12,13^ Nevertheless, despite numerous reports of successful interventions in patients with malignant diseases, there is significantly less documentation of fertility preservation in non-malignant conditions, including autoimmune diseases and SLE.^6^ From January 2007 until June 2018 (official data request from the *Ferti*PROTEKT registry by the authors and Henes et al.^6^), 146 female SLE patients with a mean age of 25.6 ± 5.8 years have been recorded in the German-speaking countries, most of them prior to chemotherapy. Used fertility-preservation measures were: cryopreservation of unfertilized oocytes after controlled ovarian stimulation (*n* = 9, six patients received additional GnRH), GnRH (*n* = 117) and cryopreservation of ovarian tissue (*n* = 29, 24 patients received additional GnRH). Only three SLE patients with cryopreserved ovarian tissue have been transplanted so far (one patient twice). A total of 163 transplantations were reported in 126 patients. Subsequently, 26 patients had 31 pregnancies, 26 of which were successfully delivered in 19 patients (6 ended in miscarriage). The success figures for cryopreservation and subsequent transplantation of ovarian tissue in postpubertal patients in the FertiPROTEKT network are comparable to the success rates in other countries.^8^ To date, more than 145 births have been achieved worldwide following orthotopic transplantation of cryopreserved ovarian tissue. The restoration rate of the endocrine function of the ovarian transplants is 85–90%, the clinical pregnancy rate is 45% and the birth rate is currently around 35–40%, although a further increase can be assumed through continued optimization of cryopreservation/thawing and surgical techniques.^8,9^ Common concerns include surgical and anaesthetic risks involved in obtaining the tissue, whereas the reintroduction of malignant cells, as discussed in the literature,^14^ is no concern in non-malignant diseases. The risk of premature menopause due to removal of one-half to two-thirds of an ovary can be considered as relatively small (∼1 year earlier after a unilateral oophorectomy).^8^ One advantage over cryopreservation of oocytes in SLE is the lack of hormonal stimulation in a phase when the disease is likely to be active. Furthermore, there is no evidence of an increased risk of twin pregnancies as opposed to embryo transfer in in vitro fertilization, which would increase the risk of an unfavourable course of pregnancy in SLE patients even further.

Regardless of the choice of fertility-preserving method, reviewing the indication for treatment with cytotoxic agents (e.g. cyclophosphamide) and, if appropriate, the regular implementation of fertility counselling for affected SLE patients is particularly important.

Our patient initially had a confirmed premature ovarian insufficiency. This was most likely therapy-related, but was potentially also favoured by her SLE. Even though the ovarian insufficiency was initially remedied by the ovarian transplant, the FSH values remained elevated at first and, subsequently, in February/March 2017 the hormonal pattern matched that of a premenopausal woman. In other cases, persisting elevation of FSH levels may be attributed to the growth of more than one follicle, resulting in fraternal twins. This phenomenon of distinct increased twin pregnancies is also observed in patients receiving assisted reproductive technology who were about to enter menopause due to their age and who also had elevated FSH levels.

In our patient, we faced a high-risk pregnancy due to her past history including relapsing lupus nephritis, cerebral ischaemia and persistent serological activity.^11^ It was therefore particularly important to harmonize the approach with the patient in a shared decision-making process by extensively discussing all facets of potential risks. While there is a high consensus regarding the safety of azathioprine and HCQ during pregnancy,^16^ the data on belimumab is sparse with only a few reported pregnancies after exposure before conception or in early pregnancy. Preliminary findings suggest that exposure early in the first trimester is unlikely to be harmful.^16^ In addition, it was unclear when conception would occur. Therefore, we agreed to continue belimumab until conception to minimize the risks of a disease flare. Despite all efforts and stable disease control of her SLE, she delivered prematurely.

The preterm birth rate in women with SLE is about twice the preterm birth rate in healthy pregnancies, and the risk of PROM is especially increased in women with active SLE and patients with renal disease.^17^ This emphasizes the need for close multidisciplinary supervision of SLE patients during pregnancy. However, twin pregnancies alone are also associated with an increased risk for PROM. An additional detrimental effect of prednisolone on preterm birth is suspected from the literature. However, our patient received only low-dose prednisolone (5 mg/day) over the preceding 18 months and no additional steroids were administered for fetal lung maturation.

In conclusion, our patient experienced a successful pregnancy after autotransplantation of cryopreserved ovarian tissue following premature ovarian failure as a result of therapy with cyclophosphamide. She was able to conceive spontaneously, and both the interventions and the course of the pregnancy proceeded without additional serious complications. We regard the procedure to be an option worthy of consideration for our predominantly young SLE patients.

## Supplemental Material

Supplemental material for Successful conception in a 34-year-old lupus patient following spontaneous pregnancy after autotransplantation of cryopreserved ovarian tissue Click here for additional data file. Supplemental Material for Successful conception in a 34-year-old lupus patient following spontaneous pregnancy after autotransplantation of cryopreserved ovarian tissue by G Chehab, J Krüssel, T Fehm, R Fischer-Betz, M Schneider, A Germeyer, MB Suerdieck, V Kreuzer and J Liebenthron in Lupus

## References

[1] Boumpas DT Austin HA Vaughan EM et al. Risk for sustained amenorrhea in patients with systemic lupus erythematosus receiving intermittent pulse cyclophosphamide therapy. Ann Intern Med 1993; 119: 366–369. 8338289 10.7326/0003-4819-119-5-199309010-00003

[2] Huong DL Amoura Z Duhaut P et al. Risk of ovarian failure and fertility after intravenous cyclophosphamide. A study in 84 patients. J Rheumatol 2002; 29: 2571–2576. 12465154

[3] Mayorga J Alpízar-Rodríguez D Prieto-Padilla J et al. Prevalence of premature ovarian failure in patients with systemic lupus erythematosus. Lupus 2016; 25: 675–683. 26678443 10.1177/0961203315622824

[4] Tamirou F Husson SN Gruson D et al. Brief report: The Euro-Lupus low-dose intravenous cyclophosphamide regimen does not impact the ovarian reserve, as measured by serum levels of anti-Müllerian hormone. Arthritis Rheumatol 2017; 69: 1267–1271. 28235250 10.1002/art.40079

[5] Oktem O Yagmur H Bengisu H et al. Reproductive aspects of systemic lupus erythematosus. J Reprod Immunol 2016; 117: 57–65. 27474801 10.1016/j.jri.2016.07.001

[6] Henes M Henes JC Neunhoeffer E et al. Fertility preservation methods in young women with systemic lupus erythematosus prior to cytotoxic therapy: Experiences from the FertiPROTEKT network. Lupus 2012; 21: 953–958. 22438026 10.1177/0961203312442753

[7] Ott J Nouri K Stögbauer L et al. Ovarian tissue cryopreservation for non-malignant indications. Arch Gynecol Obstet 2010; 281: 735–739. 19771439 10.1007/s00404-009-1224-8

[8] Beckmann M Lotz L Toth B et al. Concept paper on the technique of cryopreservation, removal and transplantation of ovarian tissue for fertility preservation. *Geburtshilfe Frauenheilkd*. Epub ahead of print 9 October 2018; 79: 53–62. 10.1055/a-0664-8619 PMC6336469 30686834

[9] Liebenthron J Montag M Reinsberg J et al. Overnight ovarian tissue transportation for centralized cryobanking: a feasible option. Reprod Biomed Online. Epub ahead of print 18 January 2019. DOI: 10.1016/j.rbmo.2019.01.006. 10.1016/j.rbmo.2019.01.006 30733076

[10] Van Vollenhoven R Voskuyl A Bertsias G et al. A framework for remission in SLE: Consensus findings from a large international task force on definitions of remission in SLE (DORIS). Ann Rheum Dis 2017; 76: 554–561. 27884822 10.1136/annrheumdis-2016-209519

[11] Andreoli L Bertsias GK Agmon-Levin N et al. EULAR recommendations for women's health and the management of family planning, assisted reproduction, pregnancy and menopause in patients with systemic lupus erythematosus and/or antiphospholipid syndrome. Ann Rheum Dis 2017; 76: 476–485. 27457513 10.1136/annrheumdis-2016-209770 PMC5446003

[12] Arbeitsgemeinschaft der Wissenschaftlichen Medizinischen Fachgesellschaften. Fertility preservation in oncological therapies: AWMF clinical practice guideline 2017 (in German), http://www.awmf.org/leitlinien/detail/ll/015-082.html (2017, accessed 19 October 2018).

[13] Von Wolff M Sänger N Liebenthron J Is ovarian tissue cryopreservation and transplantation still experimental? It is a matter of female age and type of cancer. J Clin Oncol 2018; 33: 3340–3341. 10.1200/JCO.18.00425 30289731

[14] Dolmans M-M Masciangelo R Risk of transplanting malignant cells in cryopreserved ovarian tissue. Minerva Ginecol 2018; 70: 436–443. 29644333 10.23736/S0026-4784.18.04233-8

[15] Lawrenz B Henes J Henes M et al. Impact of systemic lupus erythematosus on ovarian reserve in premenopausal women: Evaluation by using anti-Muellerian hormone. Lupus 2011; 20: 1193–1197. 21768179 10.1177/0961203311409272

[16] Flint J Panchal S Hurrell A et al. BSR and BHPR guideline on prescribing drugs in pregnancy and breastfeeding-Part I: Standard and biologic disease modifying anti-rheumatic drugs and corticosteroids. Rheumatology (Oxford) 2016; 55: 1693–1697. 26750124 10.1093/rheumatology/kev404

[17] Wei S Lai K Yang Z et al. Systemic lupus erythematosus and risk of preterm birth: A systematic review and meta-analysis of observational studies. Lupus 2017; 26: 563–571. 28121241 10.1177/0961203316686704

