# Supplementary material for: Successful conception in a 34-year-old lupus patient following spontaneous pregnancy after autotransplantation of cryopreserved ovarian tissue
Source: Lupus. 2019 Mar 24;28(5):675–80. doi: 10.1177/0961203319839482 (PMC6515711; doi:10.1177/0961203319839482)
Supplement: Supplemental material for Successful conception in a 34-year-old lupus patient following spontaneous pregnancy after autotransplantation of cryopreserved ovarian tissue [file Supplemental_Material.pdf]

Supplement to: 'Successful conception in a 34 year old lupus patient following spontaneous pregnancy after autotransplantation of cryopreserved ovarian tissue'

Gamal Chehab, Jan Krüssel, Tanja Fehm, Rebecca Fischer-Betz, Matthias Schneider, Ariane Germeyer, Moritz B. Suerdieck, Vera Kreuzer, Jana Liebenthron.

Detailed description of

- **Laparoscopical removal of ovarian tissue**
- **Transportation to the Cryobank**
- **Preparation of ovarian tissue for cryopreservation**
- **Cryopreservation protocol**
- **First quality control – testing of fresh ovarian cortex reserve**
- **Second quality control – testing of cryopreserved/ovarian cortex reserve**
- **Thawing and transplantation of ovarian tissue**

Laparoscopical removal of ovarian tissue for cryopreservation / fertility preservation

After confirming normal anatomic structures without any pathology (especially no intraabdominal adhesions, no visible endometriosis or myoma) – inspection of the ovaries showed that the right ovary did have no sign of follicular activity, whereas the left ovary gave the impression of carrying a corpus luteum. The decision was therefore made to remove tissue from the right ovary. Half of the ovary was removed with a non-cauterizing scissor, immediately extracted from the abdomen via a 10mm trocar and placed into sterile Custodiol®-medium at 4°C for further processing. Only then, the small intraabdominal bleeding of the remaining ovary was punctually cauterized. After removal of instruments and suture of the abdominal incisions, the patient was released from the hospital 4 hours after the operation.

Transportation to the Cryobank

Transportation to a nearby central university cryobank for preparation and cryopreservation was performed by overnight transportation according to Liebenthron et al.[1,2]; tissue placed in 4°C cold Custodiol® (Dr. Franz Köhler Chemie GmbH, Bensheim, Germany) solution. After arriving/overnight transportation of the tissue in special isolated and cooled transportation boxes (for 19 hours), the temperature inside of the box was optimal with 5 °C (after 19 hours).

Preparation of ovarian tissue for cryopreservation

According to Beckmann et al.[2,3] tissue preparation was performed in a sterile class II lamina air flow in a contamination-free environment. The entire ovarian piece was placed in a culture dish containing fresh Custodiol® at a convolution cooling plate (UKH602, FRYKA Kältetechnik GmbH, Esslingen, Germany) pre-cooled to a temperature of 2 °C. Using precision 22´scalpels and anatomical forceps the medulla was gently removed, leaving a tender layer of the medulla on the cortex surface to facilitate revascularization of the graft after transplantation[4]. 12 Cortex stripes (~ 8x4x1 mm) were prepared for later transplantation and cryopreserved as described below. From the remaining cortical tissue six small-standardized biopsies are obtained from different areas of the prepared

cortex using a two-mm diameter biopsy punch (PFM Medical AG, Cologne, Germany) for quality control.

Cryopreservation protocol

Cryopreservation of ovarian tissue was performed by a slow-freezing protocol modified from the procedure initially described by Gosden.[1–3,5–8] In short, ovarian tissue stripes (12 pieces and three two-mm biopsies as thawing sample) were pre-incubated for 30 minutes in 2 °C pre-cooled sterile filtered freezing solution (Leibovitz`s L-15 Medium without phenol red, Gibco by Life Technologies, Paisley, UK) containing 10% DMSO (Cryo-Sure DMSO, WAK-Chemie Medical GmbH, Steinbach, Germany) and 10% human serum albumin (HSA; Irvine Scientific, Santa Ana, CA, USA). Tissue pieces were then transferred each into one 1.8 ml cryo vial (Nunc; Thermo Fisher Scientific, Denmark) and the slow freezing process was performed in a controlled freezer (IceCube 14S-A, SY-LAB, Neupurkersdorf, Austria) which supports automatic seeding. Tissue pieces in cryo vials were cooled at a rate of -2 °C/min and automatic seeding was initiated as soon as the medium in the sample vial reached the temperature of -6 °C. After successful seeding the temperature was hold for another five to eight minutes and was then slow cooled at -0.3 °C/min to -40 °C followed by fast cooling at -10 °C/min to -140 °C. Cryo vials were plunged into liquid nitrogen and transferred into storage boxes. Storage was performed in a vapor phase storage tank (MVE 1500 Series -190°C vapour phase storage tank (Chart MVE BioMedical Industries, Inc., Garfield Heights, USA) at -190 °C.

First quality control – testing of fresh ovarian cortex reserve

Three two-mm diameter biopsies were used for the quality analysis of density and viability of primordial and primary follicles embedded in the cortical tissue[1–3] directly after transportation, preparation and before cryopreservation and three after thawing of the cryopreserved control biopsies before transplantation (thawing sample). Tissue was digested with collagenase (Sigma-Aldrich Chemie GmbH, Munich, Germany), follicles were stained with calcein–acetoxymethylester (Promega GmbH, Mannheim, Germany) and follicle density was analysed by fluorescence microscopy.[1,2,9] After counting of the fresh prepared biopsie, before cryopreservation, we analyzed viable 74 primordial follicles (PROF) and after cryopreservation (six years later) we analyzed in the same amount of tissue (three two-mm biopsies) 68 PROF.

Second quality control – testing of cryopreserved/ovarian cortex reserve

Follicle density (mean from fresh and thawed samples: 71 PROF) as well as serum anti-Müllerian (AMH) hormone concentrations 1.7 ng/ml, FSH: 5.0 mIU/ml and patient’s age at tissue removal (26 years) were regarded as the ovarian reserve parameters and were used to calculate the amount ovarian tissue required for transplantation. We decided to transplant four pieces with a size of 4x8x1 mm^3 into one peritoneal pocket near the fallopian tube.

Thawing and transplantation of ovarian tissue

The thawing protocol was modified from published procedures.[1–3,6,7,10–12] Cryo vials were kept at room temperature for 30 seconds and then immersed in a 37 °C water bath for approximately two minutes until only a centrally located thin ice spindle was left. Tissue pieces were transferred into 0,75 M sucrose (Sucrose ACS reagent, Sigma-Aldrich Chemie GmbH, Munich, Germany) in Dulbeccos phosphate buffered saline solution (DPBS CTS, Gibco by Life Technologies, Paisley, UK), containing 10% human serum albumin (HSA) (Irvine Scientific, Santa Ana, CA, USA) for 15 minutes, followed by 15 minutes incubation in 0,375 M sucrose/DPBS/HSA and another 15 minutes incubation in 0,125 M

Sucrose/DPBS/HSA. Finally, tissue was washed twice (10 and 5 min) in DPBS/HSA. The entire thawing process was performed at room temperature using an orbital shaker. Tissue was then immediately transported to the surgical theatre for transplantation.

References

1. Liebenthron J, Montag M, Reinsberg J, et al. Overnight ovarian tissue transportation for centralized cryobanking - a feasible option. *Reprod Biomed Online*. Accepted 2018. In Press.

2. Liebenthron J, Baston-Büst DM, Bielfeld AP, et al. S2k-Leitlinie: Fertilitätserhaltende Maßnahmen bei onkologischen Erkrankungen: Kryokonservierung von Keimzellen – Möglichkeiten und Techniken bei Mädchen und Frauen im fertilen Alter. *Gynäkol* 2018; 51: 926–936.

3. Beckmann M, Lotz L, Toth B, et al. Concept Paper on the Technique of Cryopreservation, Removal and Transplantation of Ovarian Tissue for Fertility Preservation. *Geburtshilfe Frauenheilkd*. Epub ahead of print 9 October 2018. DOI: 10.1055/a-0664-8619.

4. Donnez J, Dolmans M-M. Ovarian tissue freezing: current status. *Curr Opin Obstet Gynecol* 2015; 27: 222–230.

5. Gosden RG, Baird DT, Wade JC, et al. Restoration of fertility to oophorectomized sheep by ovarian autografts stored at -196 degrees C. *Hum Reprod Oxf Engl* 1994; 9: 597–603.

6. Isachenko V, Isachenko E, Reinsberg J, et al. Cryopreservation of human ovarian tissue: comparison of rapid and conventional freezing. *Cryobiology* 2007; 55: 261–268.

7. Isachenko V, Dittrich R, Keck G, et al. Cryopreservation of Ovarian Tissue: Detailed Description of Methods for Transport, Freezing and Thawing. *Geburtshilfe Frauenheilkd* 2012; 72: 927–932.

8. Bastings L, Westphal JR, Beerendonk CCM, et al. Clinically applied procedures for human ovarian tissue cryopreservation result in different levels of efficacy and efficiency. *J Assist Reprod Genet* 2016; 33: 1605–1614.

9. Liebenthron J, Köster M, Drengner C, et al. The impact of culture conditions on early follicle recruitment and growth from human ovarian cortex biopsies in vitro. *Fertil Steril* 2013; 100: 483–491.e5.

10. Rosendahl M, Schmidt KT, Ernst E, et al. Cryopreservation of ovarian tissue for a decade in Denmark: a view of the technique. *Reprod Biomed Online* 2011; 22: 162–171.

11. Dittrich R, Lotz L, Keck G, et al. Live birth after ovarian tissue autotransplantation following overnight transportation before cryopreservation. *Fertil Steril* 2012; 97: 387–390.

12. Bastings L, Liebenthron J, Westphal JR, et al. Efficacy of ovarian tissue cryopreservation in a major European center. *J Assist Reprod Genet* 2014; 31: 1003–1012.
